# Improvement of Catalytic Efficiency, Thermo-stability and Dye Decolorization Capability of *Pleurotus ostreatus* IBL-02 laccase by Hydrophobic Sol Gel Entrapment

**DOI:** 10.1186/1752-153X-6-110

**Published:** 2012-09-29

**Authors:** Muhammad Asgher, Shagufta Kamal, Hafiz Muhammad Nasir Iqbal

**Affiliations:** 1 Industrial Biotechnology Laboratory, Department of Chemistry and Biochemistry, University of Agriculture, Faisalabad, Pakistan

**Keywords:** *P. ostreatus* IBL-02, Laccase, PAGE, Sol–gel immobilization, Kinetics, Textile dye, Waste water effluent, Decolorization

## Abstract

**Background:**

In serious consideration of the worldwide environmental issues associated with the extensive use of the textile dyes and effluents generated thereof, the scientists across the world are in search for potential treatment technologies for their treatment. In such scenario the ligninolytic enzymes provide a potential alternative because they are cost effective, eco-friendly and can be applied to wide range of dye containing industrial effluents.

**Results:**

Laccase produced from *Pleurotus ostreatus* IBL-02 during decolorization of the reactive textile dye Drimarene brilliant red K-4BL (DBR K-4BL) was purified and immobilized by hydrophobic gel entrapment. The crude laccase was 4.2-fold purified with specific activity of 573.52 U/mg after passing through the DEAE-Sepharose ion exchange and Sephadex-G-100 chromatography columns. *P. ostreatus* IBL-02 laccase was found to be a homogenous monomeric protein as evident by single band corresponding to 67 kDa on native and sodium dodesylsulfate polyacrylamide gel electrophoresis (PAGE). The laccase was immobilized by entrapment in Sol–gel matrix of trimethoxysilane (T) and proplytetramethoxysilane (P) prepared using different T:P molar ratios. The free and immobilized laccases were compared to investigate the effect of immobilization on catalytic efficiency and thermo-stability features. Laccase immobilized in the Sol–gel of 1:5 T:P ratio was optimally active and thermo-stable fraction at pH 5, 60°C with half-life of 3 h and 50 min. Laccases immobilized in 1:2 and 1:5 T:P ratio gels had significantly higher *K*_m_ (83 and100mM) and *V*_max_ (1000 and 1111 mM/mg) values as compared to free laccase. After 5 h reaction time varying decolorization percentages with a maximum of 100% were achieved for different dyes and effluents.

**Conclusions:**

In summary, *P. ostreatus* IBL-02 laccase was immobilized by entrapping in a Sol–gel matrix with an objective to enhance its catalytic and stability properties. Sol–gel entrapped laccase presented potential efficiency as a biocatalyst when applied for decolorization of different dyes and effluents. The main benefits of the Sol–gel matrix immobilization processes are the eco-friendly approach, chemical free and energy saving reaction conditions.

## Background

Laccase (EC 1.10.3.2) is a blue copper oxidase secreted by white rot fungi (WRF) not only an important component of the ligninolytic enzyme system responsible for lignin degradation, but can even degrade non-aromatic compounds in the presence of low molecular weight redox mediator compounds [1]. As single enzyme and/or in combination with other ligninolytic, cellulolytic and xylanolytic enzymes, laccases have important applications in bio-ethanol production, bio-pulping in the paper and pulp industry, denim stone washing, wastewater treatment, oxidation of organic pollutants, extraction and stabilization of fruit juices, biosensor development, textile bio-finishing, beverage processing, decreasing dough extensibility in flour, animal feed, cosmetics, clinical diagnosis enzyme immunoassays, and detergent manufacturing [2-8]. However, their high production cost, low operational stabilities, availability in small amounts, susceptibility to attack by proteases and activity inhibition limit their commercial applications in industrial and environmental biotechnology [9].

Over the last few decades, intensive research in the area of enzyme technology has provided many approaches that facilitate their practical applications. The various techniques for enhancing operational stability of laccases are enzyme engineering, chemical modification, mutation and immobilization [10]. Among them, the newer technological developments in the field of immobilized biocatalysts can offer the possibility of a wider and more economical exploitation of biocatalysts in industry, waste treatment, medicine, and in the development of bioprocess monitoring devices like biosensors [11]. The method of immobilization is the most important because equipped steadiness and reusability of enzyme depend on it. Physical entrapment and surface binding are the two most commonly used methods. Entrapment is preferred over surface binding as this method is easier and cheaper, stable derivatives are formed and the structure of the enzyme remains secure [12]. A second important component of immobilization on which performance of the enzyme depends, is enzyme support. Two types of commonly used supports are hydrophobic and hydrophilic biomaterials. Hydrophobic biomaterials are preferred because these have the ability to entrap large amounts of enzyme with a much higher degree of immobilization and enzyme activity retention. The physical characteristics of Sol-gels have been extensively manipulated for enzyme immobilization and these gels have attracted the attention of biotechnologists. Sol-gels have the ability to produce enzymes in stable defined thin films that are more vigorous having ability to catalyze reactions under wide environmental conditions [13,14].

Dye containing textile waste effluents contain several types of hazardous chemicals including synthetic dyes [14]. Most of the textile industries discharged their routine waste effluents into the main water streams without or after some partial chemical / physical treatments. An eco-friendly treatment of industrial effluents is still a major environmental concern for modern world [15]. In spite of the existing physical/chemical technologies that are usually expensive and commercially or environmentally unattractive, biological processes seem as potential alternatives because they are cost effective, eco-friendly and can be applied to wide range of dye containing industrial effluents. WRF have the ability to degrade contaminants by virtue of its extracellular ligninolytic enzymes including lignin peroxidases (LiPs), manganese peroxidases (MnPs) and laccases [6,13-16]. Therefore, over the past several years, there has been great interest among researchers in the production of ligninolytic enzymes using various agro-based waste materials [5].

This manuscript describes the results of a study aimed at immobilizing a laccase, produced by the indigenous strain of *P. ostreatus* IBL-02 during decolorization of Drimarene brilliant red K-4BL [17] in Sol-gels matrix of varying hydrophobicities. The investigation also involved the comparison of kinetic, catalytic and thermo-stability properties of immobilized and free laccases and, their abilities to decolorize different textile dyes and industrial effluents.

## Results and discussion

### Source of laccase

The laccase produced by *P. ostreatus* IBL-02 during decolorization of Drimarene brilliant red K-4BL under optimum conditions [17] was used for purification, immobilization and characterization studies. Under optimum conditions, *P. ostreatus* IBL-02 produced 321 U/mL of laccase during complete decolorization (100%) of Drimarene Brilliant Red K- 4BL in 24 h. The optimum conditions were: glucose (as carbon supplement), 2 g/ 100mL; ammonium nitrate (nitrogen additive), 0.06 g/100 mL; Cu^2+^ (1mM), 1mL as metal activator; ABTS (10mM), 2mL as mediator, pH 5 and temperature, 35°C. The culture supernatant was used as crude enzyme extract for purification and immobilization purposes.

### Purification of laccase

Crude laccase was purified to homogeneity after ammonium sulfate precipitation, dialysis, DEAE-Sepharose ion exchange chromatography and Sephadex G-100 gel filtration. The four step purification protocol for laccase resulted in 4.2~fold purification (Table 1) with 11% laccase recovery. The onset of laccase precipitation occurred at 50% saturation, while complete salting out was observed at 80% saturation. The short range of precipitation i.e., from 50-80% provided evidence that there might be only one form of laccase having the same level of surface charge. After the removal of salts by dialysis, its purification factor and percent recovery were increased. Ammonium sulfate saturation to 80% resulting in 76% yield was selected for further purification. Purification of laccase after ion exchange chromatography was about 3.4 fold and its recovery was 15%. Ion exchange chromatography played an important role in the separation of the blue-colored fraction with laccase activity from the dark brown pigments. By gel filtration through a G-100 Sephadex column laccase was purified to 4.2~fold with 11% recovery.

**Table 1:** **Purification summary for laccase produced by *P*. *ostreatus *IBL-02 during decolorization of DBR K- 4BL**

**Purification steps**	**Total volume (mL)**	**Total enzyme activity (U)**	**Total protein content (mg)**	**Specific activity (U/mg)**	**% Yield**	**Purification fold**
Crude Enzyme	500	62500	2360	134.95	1	100
(NH_4_)_2_SO_4_	30	48060	138	348.26	2.5	76
Precipitation						
DEAE-Sepharose	12	9440	20.34	464.11	3.4	15
Sephadex-G-100	09	7020	12.24	573.52	4.2	11

A variety of purification techniques including, ammonium sulfate precipitation, gel filtration and ion exchange chromatography are required to purify laccase from other pigments and contaminating proteins. In line with our findings, Chen et al. [18] also reported 80% as the saturation point of ammonium sulfate for laccase isolation from *P*. *ostreatus.* Mansur et al. [19] reported that ammonium sulfate precipitation provided 57% yield with a 5~folds purification of laccase.

### Native and SDS-PAGE

**Figure 1:**
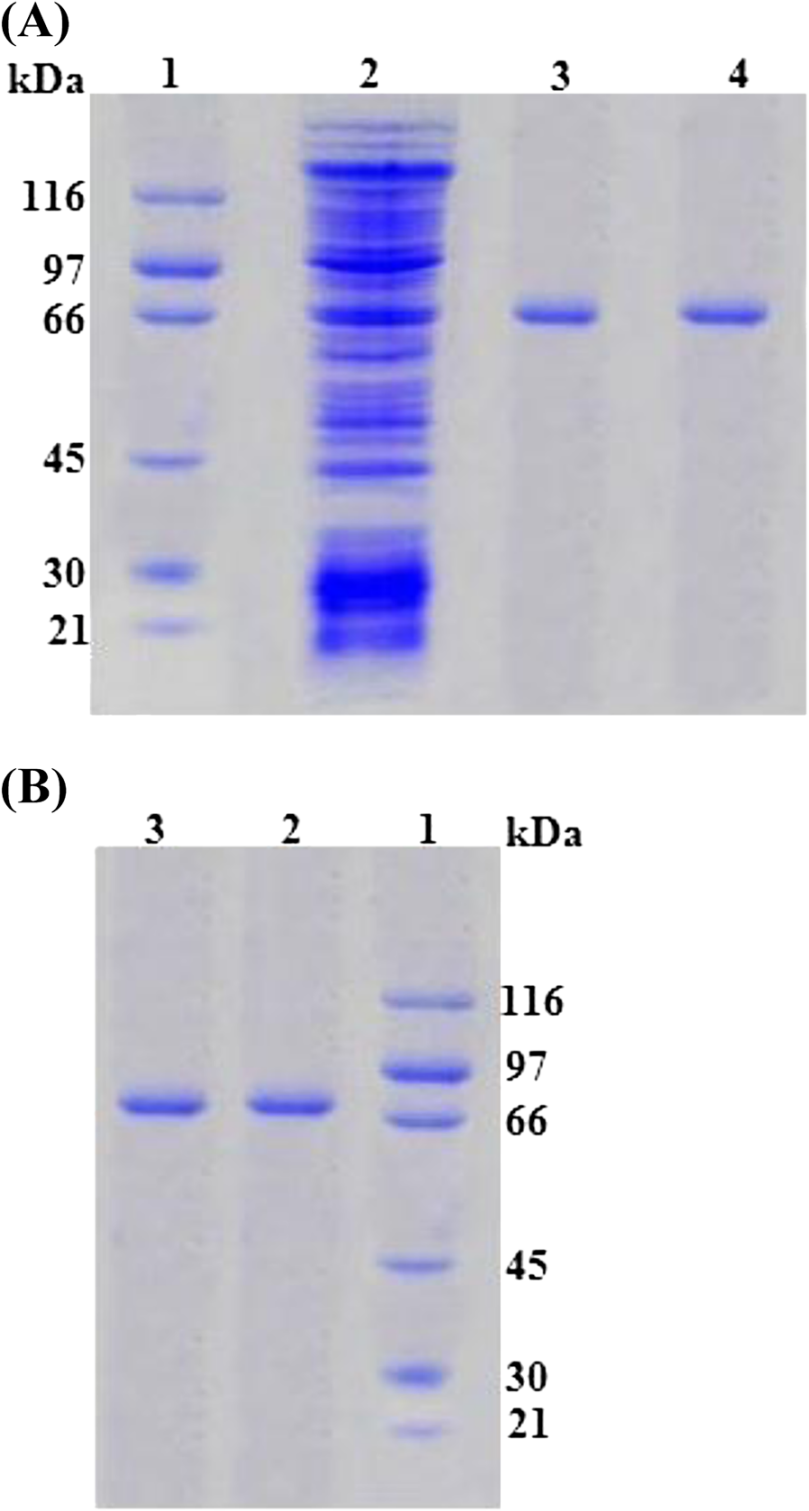
**SDS (A) and Native(B) PAGE for Laccaseproduced by *P. ostreatus *IBL-02.**

Laccase was purified to an apparent homogeneous level and the purity was confirmed on 12% native and 10% SDS-PAGE, respectively. The single band of 67 ± 1 kDa on Native and SDS-PAGE confirmed that *P. ostreatus* IBL-02 laccase was a monomeric protein (Figure 1). Similar with our findings, the laccases isolated from different strains and species of *Pleurotus* have been found to be monomeric in nature with molecular masses in 53-67kDa range [3,20]. Laccases having molecular masses of 53 KDa from *Lentinula edodes* have been reported by Nagai et al. [21]. As compared to these, laccases of much higher masses including 72 kDa from *Fusarium solani*[22] and 94 kDa for *Phanerocheate flavidoalba*[23] have also been isolated.

[Lane 1, Molecular weights in kDa of standard marker; (β-Galactosidase, 116 kDa; Phosphorylase B, 97 kDa; albumin, 66 kDa; ovalbumin, 45 kDa; carbonic anhydrase, 30 kDa and trypsin inhibitor, 21 kDa); lane 2, Crude enzyme extract; lane 3 and 4, Purified Laccase in SDS-PAGE; lane 2 and 3 in native PAGE, Purified Laccase (67 kDa)].

### Immobilization of laccase

The purified *P*. *ostreatus* IBL-02 laccase was immobilized in sol-gels of varying hydrophobicities. Specific activities of laccase immobilized in different gels are shown in (Table 2). The specific activity of immobilized laccase was higher as compared to free enzyme and it increased by increasing T:P ratio from 1:1 to 1:5 (increasing hydrophobicity of the Sol-gels). However, with increasing hydrophobic character (higher T: P ratios), the specific activity of immobilized laccase significantly decreased, possibly due to diffusion limitation barrier created by increasing the hydrophobic nature of the gel. Maximum specific activity (1326 U/mg) was observed for laccase entrapped in the Sol–gel matrix of 1:5 T:P ratio. The degree of immobilization was not dependent on the amount of protein immobilized and there was no significant effect of increasing the amount of protein on the catalytic activity of enzyme after certain limits. Smaller values of specific activity with higher degree of immobilization were due to the fact that smaller concentration of substrate could enter into the catalytic site of the enzyme. When the concentration of enzyme was high in the Sol–gel, the enzyme was present in an aggregated form rather than in a dispersed form, leading to lower activity by increasing the extent of immobilization. As laccases entrapped in 1:1, 1:2, 1:5 T:P ratio gels had higher specific activities than laccases immobilized in the rest of the gels, laccases immobilized in these gels were selected for characterization.

**Table 2:** **Activities of *P*.*ostreatus *IBL-02 laccase immobilized in sol-gels of different hydrophobicities**

**No.**	**Gel precursors TMOS:PTMS (molar ratio)**	**Specific activity (U/mg)**	**Degree of immobilization**	Specific activity corrected* (U/mg)	**Relative activity**
1	Free laccase	573		573	1
2	1:1	335	0.41	670	2.5
3	1:2	317	0.44	720	2.7
4	1:5	398	0.30	1326	3.3
5	1:10	288	0.49	587	2.0
6	1:15	170	0.52	326	1.9
7	1:20	149	0.56	266	1.7
8	1:25	97	0.58	167	1.7
9	1:30	11	0.63	17	1.5

^*^Activities were corrected with degree of immobilization.

Entrapment of laccase in Sol-gels involves adsorption phenomenon that has been reported as the best method for immobilization of laccase [24]. Previously, we [16] reported that entrapment of lignin peroxidase (LiP) from *P*. *chrysosporium* in Sol-gels caused hyper-activation but an increase in hydrophobic character above certain optimum limits caused a decrease in LiP activity. As the concentration of silane increases, the degree of immobilization also increases but the activity of enzyme decreases [25]. However, the covalent binding strategy is much more expensive because it requires glutareldehyde as coupling agent.

### Characterization of free and immobilized laccase

#### Effect of pH on free and immobilized laccase

**Figure 2:**
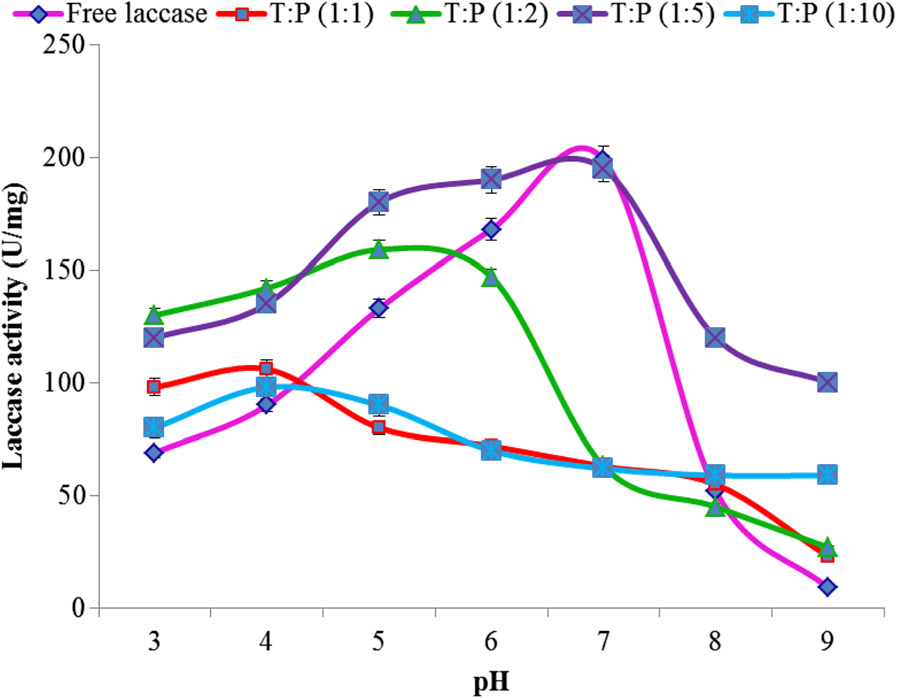
Effect of pH on activities of immobilized and free laccases.

Free and immobilized laccases showed different pH-activity profiles. The observed pH optima for free and immobilized enzymes (1:5 T:P ratio) were 6 and 4, respectively (Figure 2). Immobilization caused a decrease in optimum pH and there was a shift from less acidic towards more acidic optimum pH. All three laccases entrapped in varying T:P ratio gels showed broader pH ranges than free laccase. The method of immobilization and ionic or hydrogen bonding (secondary interaction) may be responsible for this shift in optimum pH from less acidic to more acidic. Rekuc et al. [26] also reported that after immobilization, the optimum pH of laccase shifted toward the acidic range and this shift was due to the buffering effect of the carrier surface. Contrary to our results, Qiu et al. [24] found that immobilized as well free laccase showed similar pH-activity profiles because of the clear surface of the nanoporous gold immobilization support used by them.

#### Effect of temperature on free and immobilized laccase

**Figure 3:**
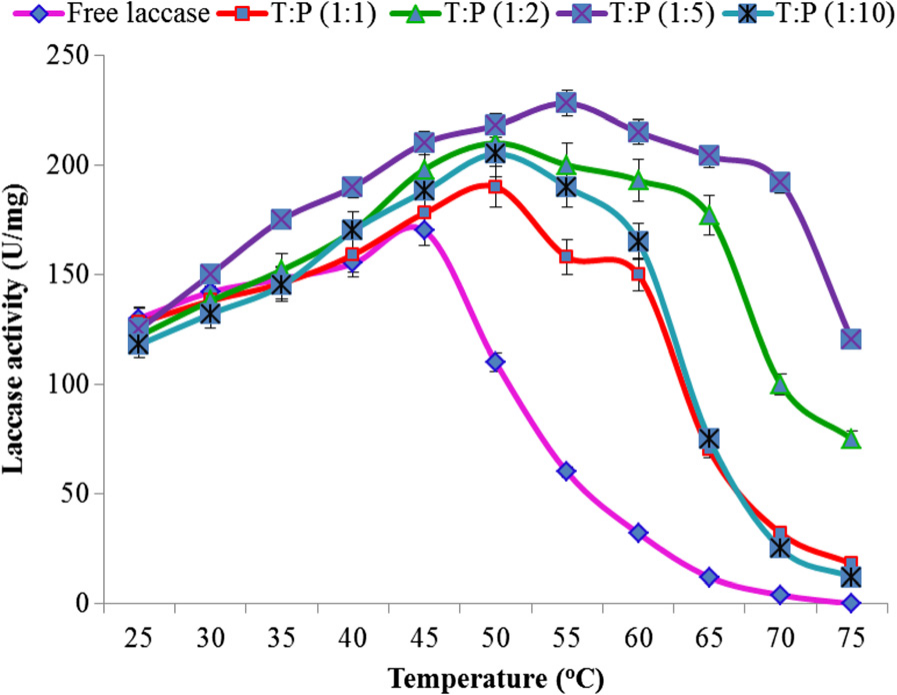
Effect of temperature on activities of immobilized and free laccases.

**Figure 4:**
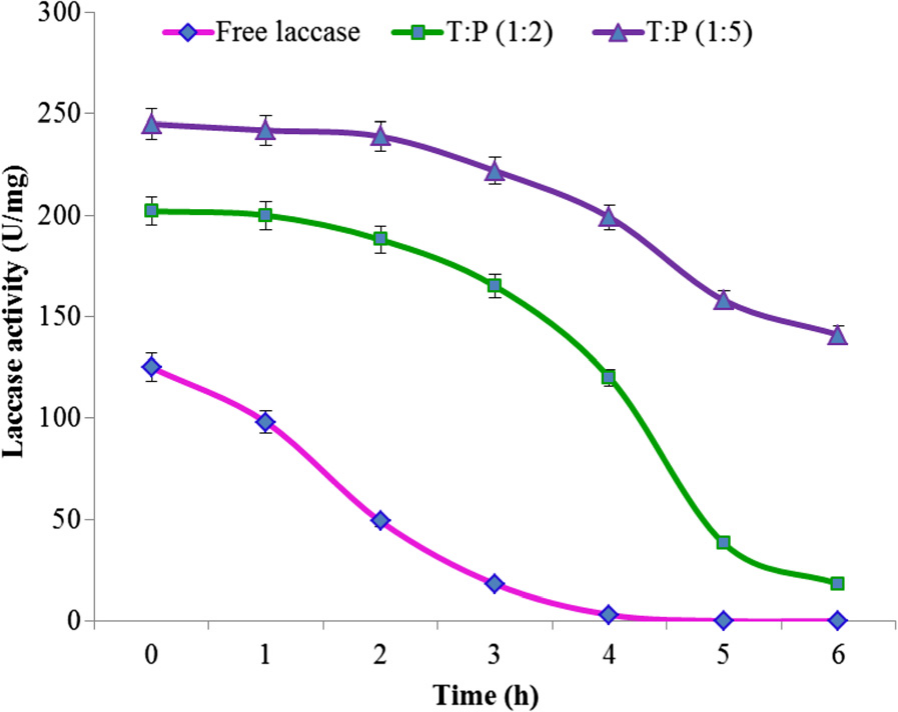
Study of half-lives of immobilized and free laccases.

Effect of temperature was studied for immobilized and free laccase in the range of 25-75°C (Figure 3). There was a shift of optimum temperature optimum from 45°C to 60°C due to immobilization and immobilized laccase was more thermos table than free laccase. Laccase entrapped in Sol–gel of 1:5 P:T ratio was the most thermos table. The order of thermo-stability was 1:5 laccase >1:2 laccase > 1:1 laccase. The immobilized laccase had a 20°C rise in optimum temperature when compared with free laccase, suggesting that immobilization of laccase in hydrophobic Sol-Gels made it more suitable candidate for industrial application. Free laccase lost its activity at temperatures above 45°C but immobilized laccase was fully active at 60°C due to the fact that external backbone of laccase became more protected and the entrapped enzyme became more resistant to heat. Half-lives at 60°C were also calculated for both entrapped and soluble lacccase as shown in Figure 4. It was approximately 3 h and 10 min for 1:2 T:P ratio and 3 h and 50 min for 1:5 T:P ratio, as compared to only 40 min for free laccase at 60°C. Laccase immobilized in 1:2 and 1:5 T:P ratios were, therefore, selected for kinetic characterization.

To make an enzyme industrially applicable, temperature is one of the most important parameters to determine the thermo-stability of the enzyme. Although it is the effect of temperature that determines which proteins lose their three dimensional structure, its effect became negligible in case of laccase due to hydrophobic interaction of laccase with the gel material that may stabilize the three dimensional structure of the enzyme. Immobilization enhances thermo-stability because maintenance of the three dimensional structures of protein competes with denaturation and loss of catalytic activities of the proteins [27]. Arica et al. [28] reported that immobilized laccase lost its activity at a mud slower rate than free enzyme by the rise in temperature. In line with our findings, immobilized laccase has been reported to withstand a wider range of temperatures (50~80°C) than free laccase [29]. Immobilization probably prevents unfolding of laccase that results in a longer half-life as compared to free laccase [30].

#### Effect of substrate concentration: Determination of K_m_ and V_max_

**Figure 5:**
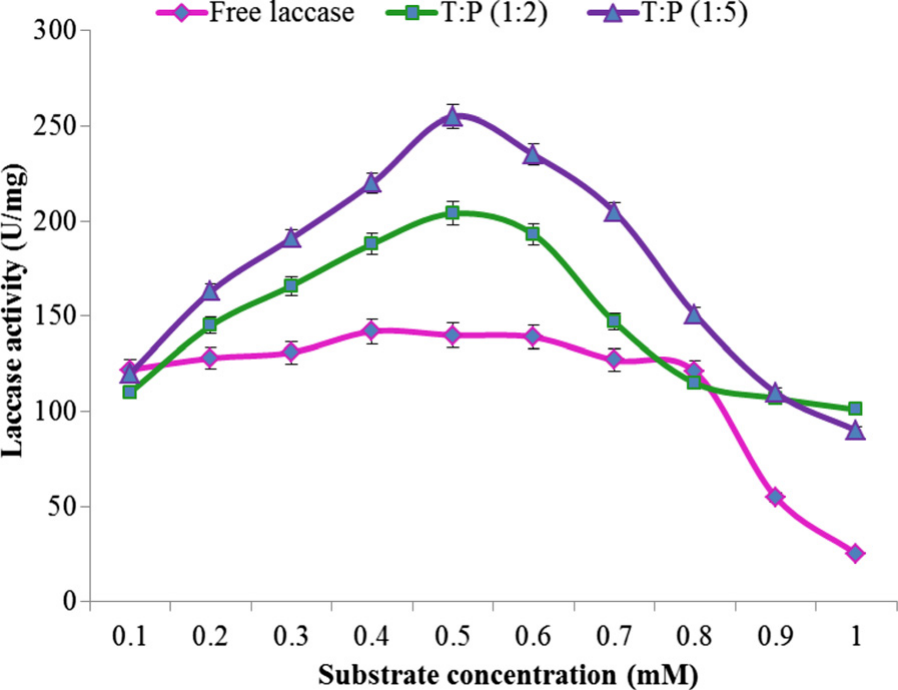
Effect of substrate concentration on free and immobilized laccase.

Kinetics of entrapped/free laccase was found to be affected by the rate of diffusion of ABTS to the enzyme active site entrapped in the Sol–gel matrices of various hydrophobicities. Results shown in (Figure 5) indicated that laccases entrapped in 1:2 and 1:5 T:P ratio gels had more affinity for ABTS but their interaction decreases with further increase in gel hydrophobicity. It was noticed that entrapped laccase was highly active having higher V_max_ values with lower ABTS concentrations range as compared to free laccase, but the reaction rate decreased with high substrate concentrations. Higher substrate concentrations may have created steric hindrance in the access of the substrate to the enzyme active site due to diffusion limitations of the substrate into the gel matrix.

**Figure 6:**
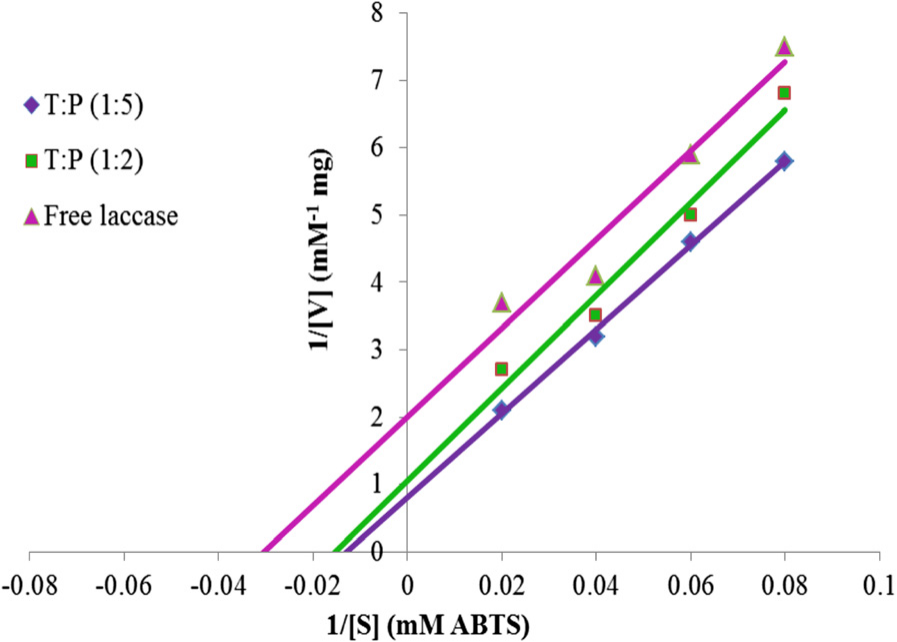
**Lineweaver-Burk reciprocal plot: Determination of *K***_**m**_**and *V***_**max**_**for free and immobilized Laccase.**

Classical Lineweaver-Burk plot transformation of the Michaelis-Menten rate equation was used to determine the kinetic parameters for free and immobilized laccases using ABTS as substrate. The effect of immobilization on diffusion of substrate and product formation was determined using effectiveness factor (EF). *K*_m_ and *V*_max_ values were calculated by intercepting the line on the X-axis and Y-axis using ABTS as substrate. Immobilized laccase had a higher *K*_m_ value than soluble laccase (Figure 6). Laccase in 1:2 and 1:5 T:P ratios had 83 and 100 mM value of *K*_m_ respectively as compared to 33 mM for free laccase. The EF value of 16 (lower than soluble) indicated that there was negligible diffusion of substrate and product removal from the gel-entrapped laccases. Immobilization offered resistance to diffusion and increased the capacity to capture a high concentration of product inside the gel. However, it could be inferred that low diffusion of substrate was the major cause of the high *K*_m_ and immobilization hindered the conformational change of catalyst that was also shown by the high *V*_max_. Furthermore, lower *K*_m_ for substrate indicated that it had high affinity for its substrate and lower value of *V*_max_ indicated that a small amount of enzyme can convert substrate into the product. Catalytic activity for free and immobilized laccase was 0.92 S^-1^ M^-1^, 0.85 S-1 M^-1^ for 1:2, 1:5 T:P ratio and 1.27 S^-1^ M^-1^ for free laccase respectively.

Although immobilized laccases have higher *K*_m_ values than soluble counterparts, the immobilized laccase have been proved to have excellent reusability, thermal constancy and equipped permanence due to high *V*_max_[24]. The *K*_m_ value indicates the interaction of enzyme with its substrate. Rekuc et al. [26] immobilized laccase in cellular foams and found that *K*_m_ for the soluble and entrapped laccase were 39.4, 133.4 and the *K*_cat_ for free and immobilized laccase was 86 and 117. Wang et al. [31] reported that laccase, after immobilizing in silica nano-particles, showed 3.28 mM and 155.4 min^-1^, *K*_m_ and *K*_cat_ respectively.

### Decolorization of Textile dyes and effluents

**Figure 7:**
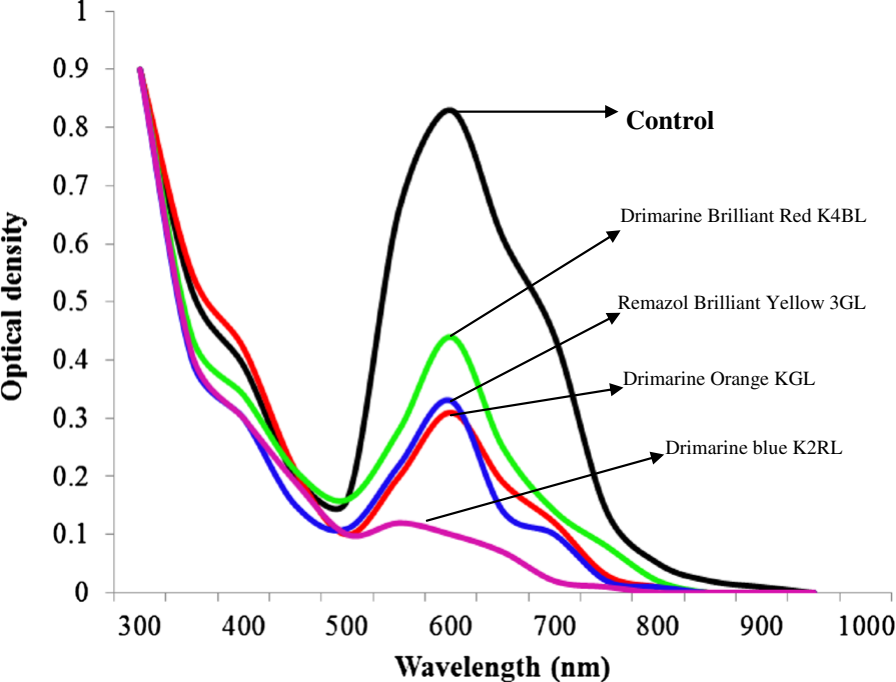
UV–vis absorption spectra of textile dyes obtained after 5h treatment with free laccase.

**Figure 8:**
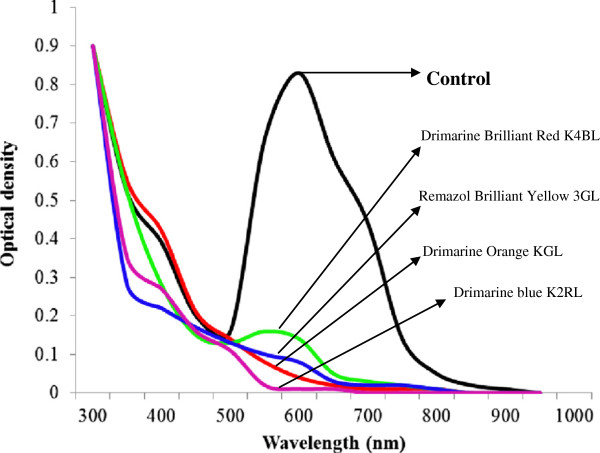
UV–vis absorption spectra of textile dyes obtained after 5h treatment with immobilized laccase.

**Figure 9:**
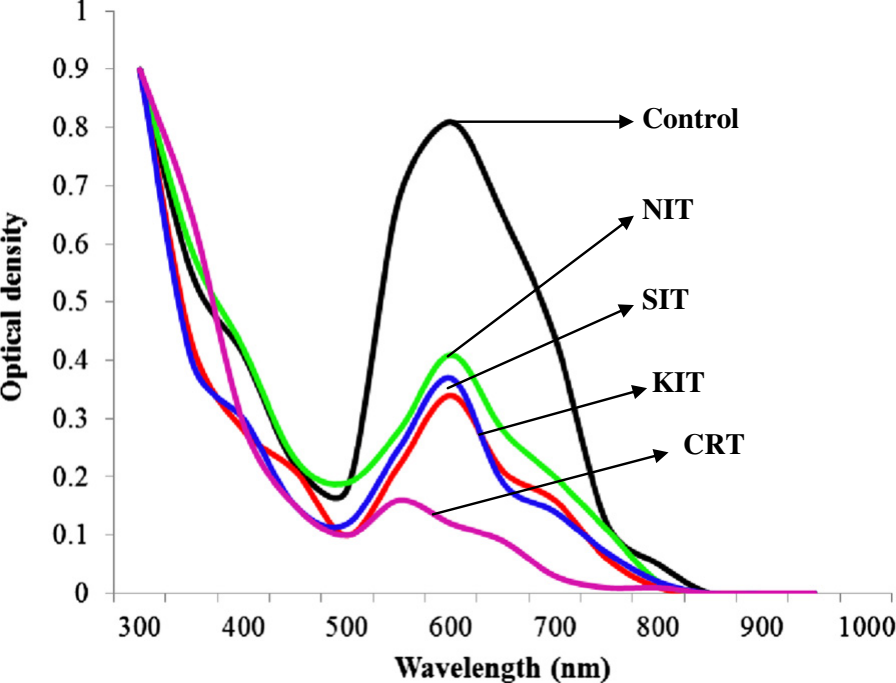
UV–vis absorption spectra of textile effluents obtained after 5h treatment with free laccase.

**Figure 10:**
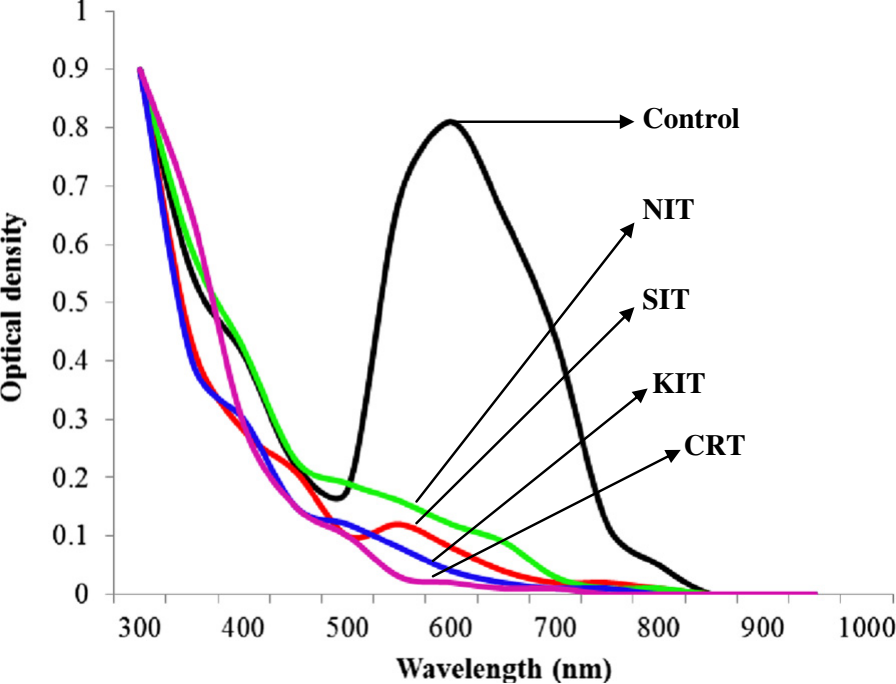
UV–vis absorption spectra of textile effluents obtained after 5h treatment with immobilized laccase.

Free and immobilized laccases from *P. ostreatus* showed different decolorization profiles for different dye-stuffs. It was noted that immobilized laccase was more efficient decolorizer of all reactive dyes as compared to its free counterpart. The best decolorization results (100%) were observed for Drimarine blue K2RL (Figures 7 and 8). In case of industrial effluents the CRT effluent was completely decolorized by immobilized laccase, followed by 97 % decolorization of SIT effluent after 5 h of incubation with ABTS as mediator (Figures 9 and 10). The decolorization of other effluents was also significantly higher for immobilized laccase as compared to free enzyme and ABTS was found to enhance the dye decolorization capacities of both free and immobilized laccase.

Laccases have been considered as the major dye degrading enzymes and are efficient decolorizers of dyes present in industrial wastewaters [32]. The ability of laccases to decolorize dye containing effluents is correlated to its ability to degrade different dyes present in the effluent. Immobilization modifies the activity, selectivity, and equipped permanence of enzymes. Immobilized laccases are more robust dye degraders as compared to their free counterparts [33]*.* The variation in effluent composition is responsible for the difficulty of its decolorization by enzyme extracts from different fungi [34]. The present sol–gel matrix-entrapped laccase seems to have potential capabilities to meet the challenges of modern industrial sector, especially for bioremediation in the textile industry.

## Conclusions

Laccase isolated from an indigenous fungal strain *P. ostreatus* IBL-02 was immobilized by Sol–gel entrapment with an objective to improve its catalytic and thermo-stability and reusability. Hydrophobic gel entrapment resulted in increased half-life, *V*_max_ and *K*_m_ values for the enzyme that may be desirable characteristics for its industrial applications. Moreover, the Sol–gel entrapped laccase presented potential efficiency as a biocatalyst for the decolorization of different dyes and local textile wastewaters. The main benefits of the Sol–gel matrix immobilization processes are the eco-friendly approach, chemical free and energy saving reaction conditions. In view of the long term trend of striving for more environmental friendly industrial processes, the health concerns regarding harmful chemicals, the versatility, non-toxicity and mild reaction conditions, the Sol–gel immobilization technology is likely to remain the subject of intensive research investigations in different sectors of industrial and environmental biotechnology.

### Experimental

#### Chemicals

Trimethoxysilane, proplytetramethoxysilane, polyvinyl alcohol, Sephadex G-100, 2, 2′-azino-bis-3-ethylbenzothiazoline-6-sulfonic acid (ABTS), Coomassie Brilliant Blue G-250, sodium dodecylsulphate (SDS), trizma base and standard Protein markers were purchased from Sigma-Fluka-Aldrich (USA). All other chemicals were of analytical grade and were mainly purchased from Merck (Germany) and Scharlau (Spain). For decolorization studies, four different dyes and textile industry effluents were collected onsite from local textile industries in Faisalabad, Pakistan.

#### Source of laccase

In a previous study, an indigenous novel strain *P. ostreatus* IBL-02 was found to produce substantial amount of laccase as a major enzyme during decolorization of the reactive textile dye Drimarene Brilliant Red K- 4BL. For maximum laccase production and dye decolorization, the physical and nutritional growth conditions have also been optimized [17]. Laccase was therefore produced under pre-optimized conditions for purification and immobilization studied.

#### Laccase activity assay

Laccase activities in the collected samples were determined at room temperature by the UV/Vis spectrophotometric assay [8]. The activity of laccase was determined by monitoring the ABTS oxidation in a reaction mixture containing 1ml of 1mM ABTS and 1ml of 50mM malonate buffer (pH 4.5) and 100μL of culture supernatant. The reaction mixture was incubated at 25°C and absorbance of each sample was taken at 420nm after 10 min. Blanks contained 100μL of distilled water instead of enzyme solution or culture supernatant. Laccase activity was expressed as international units (IU) and defined as the amount of enzyme forming 1 μmol of ABTS·+ per min under the assay conditions.

#### Protein estimation

Proteins were estimated using the Bradford micro assay [35] using bovine serum albumin (BSA) as standard. To 1mL of Bradford reagent, 100μL of each solution were added and mixed on a vortex mixer. The reagent blank was run by adding 100μL of distilled water to 1mL of the Bradford reagent. The change in absorbance (ΔA) at 595nm for all samples was determined within 15–30 min.

#### Purification of laccase

The culture filtrate was first filtered and centrifuged at 3000 × g and supernatant was then subjected to ammonium sulfate precipitation. The precipitate obtained was dialyzed and lyophilized and then loaded onto a DEAE-Sepharose anion-exchange column 1.5 × 18 cm, equilibrated with 10 mM sodium acetate buffer (pH 4.5), with a linearly increasing NaCl concentration gradient (0 to 0.5 μM) in the same buffer. The six fractions containing laccase activity were pooled, concentrated, and dialyzed overnight against same buffer. Gel filtration chromatography was performed using sephadex G-100 column 2.0 × 40 cm. The DEAE-purified sample was loaded on to the column and 3 mL fractions were collected. The eluted active fractions were dialyzed and protein content was determined by Bradford method.

#### Native and SDS-PAGE

The purified and lyophilized sample was dissolved in a minimum amount of 50mM malonate buffer, and subjected to 12% native PAGE and 10% SDS-PAGE using a Vertical Minigel electrophoresis apparatus (V-GES, Wealtec Corporation, U.S.A) to determine sample purity and approximate mass of laccase [36]. The approximate molecular mass of the laccase was determined after gel staining with Coomassie Brilliant Blue G followed by the calibration against broad-range molecular weight markers (Sigma, USA), which contained proteins ranging from 21–116 kDa.

### Preparation of hydrophobic gels and immobilization of laccase

To prepare the Sol–gel thin films for enzyme entrapment purposes, TMOS and PTMS were used in different molar TMOS: PTMS (T: P) ratios by adopting the methodology as described earlier by Asgher et al. [10]. TMOS and PTMS were used in molar T: P ratios of 1:1, 1:5, 1:10, 1:15, 1:20 and 1:25 to prepare gels of different hydrophobicity, in ascending order. Laccase isolated from *P*. *ostreatus* IBL-02 was suspended in water (12.5 mg/mL), shaken for 5 min and centrifuged. The supernatant fluid (400 μL) was added to a mixture of polyvinyl alcohol and water. The solution was shaken and PTMS was added, followed by TMOS. The reaction mixture was vigorously shaken for 5 sec on a vortex mixer and then gently shaken by hand. After about 30 sec, when the mixture formed a clear homogenous solution, it was placed in an ice bath until gel formation occurred. Laccase activity and protein contents of entrapped enzymes (in gels of different hydrophobicity) were determined. The entrapped enzymes having highest specific enzyme activity and protein contents were selected for further characterization.

### Characterization/Comparison of free and immobilized laccases

Purified native and Sol–gel entrapped laccases were characterized to determine and compare their pH and temperature optima, and kinetic constants such as *V*max (maximum rate), *K*m (Michaelis constant) and *K*cat (catalytic efficiency).

#### Effect of immobilization on optimum pH

The activities of purified native and entrapped laccases were studied over a pH range of 2.0-10.0 using ABTS as substrate. The buffers (0.1M) used were: pH 2–2.8, tartaric acid/sodium tartrate; pH 3–3.6, glycine/HCl); pH 3.8-4.5, glutamic acid/HCl; pH4.6-6.0 sodium acetate/acetic acid; pH 6–7, sodium phosphate; pH 7.5-8, Tris–HCl; and pH 9–10, glycine-NaOH buffer.

#### Effect of immobilization on optimum temperature and thermo-stability

The temperature activity profile of free and entrapped laccase was determined at different temperatures ranging from 25-75°C for 30 min. For determination of half-lives the enzymes were incubated at 60°C for varying time periods before carrying out standard laccase assay.

#### Effect of immobilization on kinetic constants Km, Vmax and Kcat

The Michaelis-Menten kinetic constants including *K*_m_, *V*_max_*and K*_cat_ were determined by using varying concentrations of ABTS ranging from 0.1-1 mM. Laccase activity was determined for each concentration of ABTS keeping enzyme concentration constant. Lineweaver-Burk plots were constructed between reciprocals of the initial reaction rates (1/V^o^) and varying substrate concentrations [1/S].

### Applications of free and immobilized laccase

#### Decolorization of dyes

Free and immobilized laccases were used for the decolorization of four reactive textile dyes (Drimarine Blue K2RL; Drimarine Orange KGL; Drimarine Brilliant Red K4BL and Remazol Brilliant Yellow 3GL). The working conditions of a single continuous operation were: two parallel batch of triplicate flasks containing 10 mL of free and 5 g of sol–gel-entrapped biocatalyst (laccase) was transferred to 100 mL of 0.01 % individual dye solutions with 1mL of 1mM ABTS as a laccase mediator followed by the incubation at 25°C for 5 h in rotary shaker (120 rpm). The culture supernatants recovered after filtration and centrifugation of the enzyme treated samples collected after every each hour were subjected to the residual dyestuff analysis. Absorbance measurements were done by a UV-Visible spectrophotometer (T-60, PG instruments, UK). The absorbance values for respective supernatants at each time period were corrected by subtracting the values for respective control fraction (containing only the reaction medium without enzyme). Decolorization of dye solution was determined by a reduction in optical density at the wavelength of maximum absorbance at λmax (590 nm) by UV–vis spectrophotometric spectrum.

#### Decolorization of real textile industry effluents

Different dye containing practical textile industry effluents of different colors were collected from Sitara textile (SIT), Nishat textile (NIT), K&N textile (KNT) and Crescent textile (CRT) units of Faisalabad. The effluent source industries did not disclose the names and structures of dyes being used due to their business secrets. The working conditions of a single continuous operation were: two parallel batches of triplicate flasks containing 10 mL of free and 5 g of sol–gel-entrapped laccase, respectively, 100 mL of individual dye solutions/textile effluents with 1 mL of 1 mM ABTS as laccase mediator, and incubated at 30°C in shaking incubator (120 rpm) for 5 h reaction time. The samples collected at one h intervals from each flask were used to determine the percentage color removal of textile dyes and effluents by considering the initial and final effluent absorbance. All the collected samples were centrifuged at 5,000×g for 15 min at room temperature (25°C) and clear supernatants were analyzed spectrophotometrically. Decolorization of individual effluents was determined by a reduction in optical density at the wavelength of maximum absorbance (λmax).

## Competing interests

The authors have no competing interests.

## Authors’ contributions

HMNI (Research Associate of the project) and SK (PhD Research Student) participated in carried out the experimental work on microbial cultivation, laccase production, extraction, purification, Sol–gel immobilization and kinetic characterization of free and immobilized Laccase. They also participated in drafting the manuscript. All the research work was carried out under the supervision of MA (Principal Investigator of the project), who designed the project and supervised all the experimental and analytical work. All authors read and approved the manuscript before submission.

## References

[B1] Wesenberg D Kyriakides I Agathos SN White-rot fungi and their enzymes for the treatment of industrial dye effluents Biotechnol Adv 2003 22 161 187 10.1016/j.biotechadv.2003.08.011 14623049

[B2] Colao MC Lupino S Garzillo AM Buonocore V Ruzzi M Heterologous expression of lcc1 gene from Trametes trogii in Pichia pastoris and characterization of the recombinant enzyme Microb Cell Fact 2006 5 31 10.1186/1475-2859-5-31 17038162 PMC1618855

[B3] Asgher M Bhatti HN Ashraf M Legge RL Recent developments in biodegradation of industrial pollutants by white rot fungi and their enzyme system Biodegradation 2008 19 771 783 10.1007/s10532-008-9185-3 18373237

[B4] Kim J-M Park S-M Kim D-H Heterologous expression of a tannic acid-inducible laccase3 of Cryphonectria parasitica in Saccharomyces cerevisiae BMC Biotechnol 2010 10 18 10.1186/1472-6750-10-18 20178646 PMC2839966

[B5] Stoilova I Krastanov A Stanchev V Properties of crude laccase from Trametes versicolor produced by solid-substrate fermentation Adv Biosci Biotechnol 2010 1 208 215 10.4236/abb.2010.13029

[B6] Asgher M Iqbal HMN Characterization of a novel manganese peroxidase purified from solid state culture of Trametes versicolor IBL-04 BioRes 2011 6 4317 4330

[B7] Reiss R Ihssen J Thöny-Meyer L Bacillus pumilus laccase: a heat stable enzyme with a wide substrate spectrum BMC Biotechnol 2011 11 9 10.1186/1472-6750-11-9 21266052 PMC3041658

[B8] Asgher M Iqbal HMN Asad MJ Kinetic characterization of purified laccase produced from Trametes versicolor IBL-04 in solid state bio-processing of corncobs BioRes 2012 7 1171 1188

[B9] Kunamneni A Camarero S García-Burgos C Plou FJ Ballesteros A Alcalde M Engineering and Applications of fungal laccases for organic synthesis Microb Cell Fact 2008 7 32 10.1186/1475-2859-7-32 19019256 PMC2613868

[B10] Asgher M Iqbal HMN Irshad M Characterization of purified and Xerogel immobilized Novel Lignin Peroxidase produced from Trametes versicolor IBL-04 using solid state medium of corncobs BMC Biotechnol 2012 12 46 10.1186/1472-6750-12-46 22862820 PMC3442999

[B11] Cheng J Randall A Baldi M Prediction of Protein Stability Changes for Single-Site Mutations Using Support Vector Machines Prot Str Func Bioinf 2006 62 1125 1132 10.1002/prot.20810 16372356

[B12] Almeida VM Branco CRC Assis SA Vieira IJC Braz-Filho R Branco A Synthesis of naringin 6"-ricinoleate using immobilized lipase Chem Central J 2012 6 41 10.1186/1752-153X-6-41 PMC3374675 22578215

[B13] Iqbal HMN Asgher M Characterization and decolorization applicability of xerogel matrix immobilized manganese peroxidase produced from Trametes versicolor IBL-04 Protein Pept Lett 2012 In-Press, PPL-EPUB-20120925-3 10.2174/0929866511320050013 23016633

[B14] Irshad M Bahadur BA Anwar Z Yaqoob M Ijaz A Iqbal HMN Decolorization applicability of sol–gel matrix-immobilized laccase produced from Ganoderma leucidum using agro-industrial waste BioRes 2012 7 3 4249 4261

[B15] Saratale RG Saratale GD Chang JS Govindwar SP Outlook of bacterial decolorization and degradation of azo dyes: a review J Taiwan Inst Chem Eng 2011 42 138 157 10.1016/j.jtice.2010.06.006

[B16] Asgher M Asad MJ Bhatti HN Legge RL Hyperactivation and thermo-stabilization of Phanerochaete chrysosporium lignin peroxidase by immobilization in xerogels World J Microbiol Biotechnol 2007 23 525 531 10.1007/s11274-006-9255-9

[B17] Kamal S Asgher M Khalil-ur-Rehman Zahir ZA Hyperproduction of laccase by Pleurotus ostreatus IBL-02 during decolorization of drimarene brilliant red K-4BL Fresen Environ Bull 2011 20 1478 1486

[B18] Chen S Ge W Buswell JA Biochemical and molecular characterization of a laccase from the edible straw mushroom Volvariella volvacea Eur J Biochem 2004 271 318 328 10.1046/j.1432-1033.2003.03930.x 14717699

[B19] Mansur M Arias ME Patino JLC Gonzalez MFAE The white-rot fungus Pleurotus ostreatus secretes laccase isozymes with different substrate specificities Mycologia 2003 95 1013 1020 10.2307/3761909 21149010

[B20] Miao L Zhang G Wang H Ng T Purification and Characterization of a Laccase from the edible wild mushroom Tricholoma mongolicum J Microbiol Biotechnol 2010 20 1069 1076 10.4014/jmb.0912.12033 20668399

[B21] Nagai M Sato T Saito K Kawata M Purification and characterization of an extracellular laccase from the edible mushroom Lentinula edodes, and decolorization of chemically different dyes Appl Microbiol Biotechnol 2002 60 327 335 10.1007/s00253-002-1109-2 12436315

[B22] Du Z Sun XB Purification and characterization of laccase from Curvularia trifol Adv Mat Res 2010 116 2215 2219

[B23] Perez J Rubia TD Hamman OB Martinez J Phanerochaete flavidoalba laccase induction and modification of manganese peroxidase is enzyme pattern in decolorized olive oil mill wastewaters Appl Environ Microbiol 1998 64 2722 2729 10.1128/aem.64.7.2726-2729.1998 PMC106454 9647858

[B24] Qiu H Xu C Huang X Ding Y Qu Y Gao P Immobilization of laccase on nanoporous gold: comparative studies on the immobilization strategies and the particle size effects J Phys Chem 2009 113 2521 2525

[B25] Clifford JS Legge RL Use of water to evaluate hydrophobicity of organically-modified Xerogel enzyme supports Biotechnol Bioeng 2005 92 231 237 10.1002/bit.20595 15988768

[B26] Rekuc A Bryjak J Szymanska K Jarzebski AB Laccase immobilization on mesostructured cellular foams afford preparations with ultra-high activity Proc Biochem 2009 44 191 198 10.1016/j.procbio.2008.10.007

[B27] Huang J Liu Y Wang X Silanized palygorskite for lipase immobilization J Mol Catal B: Enz 2009 57 10 15 10.1016/j.molcatb.2008.06.009

[B28] Arica M Altıntas B Bayramoglu G Immobilization of laccase onto spacer-arm attached non-porous poly(GMA/EGDMA) beads: Application for textile dye degradation Biores Technol 2009 100 665 669 10.1016/j.biortech.2008.07.038 18768310

[B29] Singh G Bhalla A Capalash N Sharma P Characterization of immobilized laccase from γ-proteobacterium JB: Approach towards the development of biosensor for the detection of phenolic compounds Indian J Sci Technol 2010 2 48 53

[B30] Prasad KK Mohan SV Bhaskar YV Ramanaiah SV Babu VL Pati BR Sarma PN Laccase production using Pleurotus ostreatus 1804 immobilized on PUF cubes in batch and packed bed reactors: influence of culture conditions J Microbiol 2005 43 301 307 15995650

[B31] Wang F Guo C Yang L Liu CZ Magnetic mesoporous silica nanoparticles: Fabrication and their laccase immobilization performance Biores Technol 2010 101 8931 8935 10.1016/j.biortech.2010.06.115 20655206

[B32] Pazarlioglu NK Sariisik M Telefoncu A Laccase production by Trametes versicolor and application to denim washing Proc Biochem 2005 40 1673 1678 10.1016/j.procbio.2004.06.052

[B33] Bayramoglu G Yilmaz M Arica MY Reversible immobilization of laccase to poly(4-vinylpyridine) grafted and Cu(II) chelated magnetic beads: Biodegradation of reactive dyes Biores Technol 2010 101 6615 6621 10.1016/j.biortech.2010.03.088 20388589

[B34] Maas R Chaudhari S Adsorption and biological decolorization of azo dye reactive red 2 in semicontinuous anaerobic reactors Proc Biochem 2005 40 699 705 10.1016/j.procbio.2004.01.038

[B35] Bradford MM A rapid and sensitive method for quantification of microgram quantities of protein utilizing the principle of protein dye binding Anal Biochem 1976 72 248 254 10.1016/0003-2697(76)90527-3 942051

[B36] Laemmli UK Cleavage of structural proteins during assembly of head of bacteriophage T4 Nature 1970 227 680 685 10.1038/227680a0 5432063

